# Invalid Nucleic Acid Test Results May Limit Deceased Donor Organ Utilization

**DOI:** 10.1111/tid.70247

**Published:** 2026-05-22

**Authors:** Sara Dionne, Hillary Akana, Kaitlin Logue, Brenden O'Neale, Chris Curran, Sean VanSlyck

**Affiliations:** ^1^ Eurofins Donor & Product Testing, LLC Centennial Colorado USA; ^2^ New England Donor Services Waltham Massachusetts USA; ^3^ Sierra Donor Services Sacramento California USA

**Keywords:** donor eligibility, donor screening, infectious disease, nucleic acid testing, organ transplantation

## Abstract

**Background:**

Nucleic acid testing (NAT) is essential for detecting transmissible viral infections in deceased organ donors and guiding safe organ allocation. However, invalid discriminatory NAT (dNAT) results may lead to unnecessary donor exclusion and reduced organ availability.

**Methods:**

We performed a retrospective analysis of HIV‐1/2, HBV, and HCV NAT results from deceased organ donors in the United States. Donor specimens were tested at eleven laboratories using FDA‐approved donor screening assays. Donor serologic results and organ utilization outcomes were reviewed to evaluate the clinical impact of invalid dNAT results.

**Results:**

Invalid dNAT results occurred in 0.027% of donor cases (*n* = 27), predominantly in cases suggestive of HCV infection (*n* = 22). Although infrequent, these cases had a disproportionate impact on organ utilization. Serologic findings frequently supported true infection despite unresolved NAT results. Repeat testing did not resolve these cases and extended result reporting times by a median of 22 h. In 30% of cases (*n* = 6) in which organ utilization was available, organs were not transplanted, reflecting uncertainty in infectious risk assessment.

**Conclusion:**

Although rare, invalid dNAT results have clinical and operational consequences, contributing to delays, conservative donor classification, and reduced organ utilization. These findings highlight the need for standardized reporting of invalid results and access to rapid alternative confirmatory testing to support informed clinical decision‐making and reduce unnecessary organdiscard.

## Graphical abstract

Rare instances occur in which discriminatory NAT results cannot be resolved in deceased organ donor cases. When these events occur the impact on organ donation can be substantial.

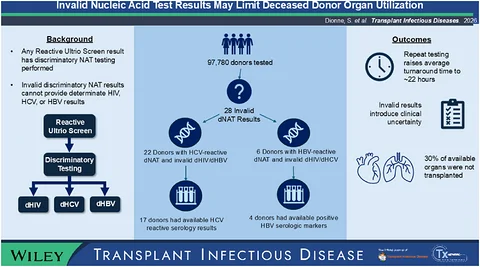

**Abbreviations**
dNAT: discriminatory NAT
FDA: Food and Drug Administration
HIV: human immunodeficiency virus
HBV: hepatitis B
HCV: hepatitis C
anti‐HCV: hepatitis C antibody
HBsAg: hepatitis B surface antigen
anti‐HBc: hepatitis B core total antibody
NAT: nucleic acid testing
OPTN: Organ Procurement and Transplantation Network
OPOs: organ procurement organizations
PHS: Public Health Service
qPCR: quantitative polymerase chain reaction
TMA: transcription‐mediated amplification

## 1 Introduction

As the demand for transplantable organs continues to exceed supply, transplant programs increasingly rely on donors with infectious risk factors, including those with hepatitis C (HCV) or hepatitis B (HBV) infection. Advances in antiviral therapy have substantially improved posttransplant outcomes, allowing broader utilization of organs from viremic donors. In this context, accurate and timely infectious disease testing is essential not only for recipient safety but also for informed organ allocation and acceptance decisions.

Nucleic acid testing (NAT) detects targeted nucleic acid sequences in blood specimens with high sensitivity and specificity and is used for screening donations of blood products and donors of organs, tissues, corneas, and cell‐based products [1]. In the deceased organ donor screening, NAT enables rapid detection of early, active viral infections by identifying viral RNA or DNA, thereby shortening the diagnostic window period for infections such as HIV, HBV, and HCV that may not yet be detectable by serologic screening tests [2, 3]. By identifying infections earlier, NAT reduces the risk of donor‐derived viral transmission to immunosuppressed recipients [4, 5] and supports more precise risk stratification during organ allocation. Even when NAT results are reactive, this information facilitates informed allocation decisions and plan posttransplant management. NAT enhances both safety and organ utilization in the deceased donor setting.

The US Public Health Service (PHS) provides recommendations to minimize the risk of HIV, HBV, and HCV transmission in organ transplantation [2]. NAT testing for HIV1/2, HBV, and HCV is required for organ allocation in the United States [6]. In this study, NAT was performed using transcription‐mediated amplification (TMA). When an initial NAT screen was reactive, discriminatory testing was subsequently performed to identify the specific virus or viruses detected. If the discriminatory testing yields invalid results, the presence or type of viral infection cannot be reliably determined.

Current organ allocation systems rely on qualitative infectious disease results reported as reactive or nonreactive and do not capture assay limitations or unresolved testing scenarios. When discriminatory NAT results are invalid, organ procurement organizations (OPOs) may conservatively classify donors as reactive for multiple viruses, even when serologic and clinical data suggest otherwise. This approach is applied by OPOs given the indeterminate results and inability to specifically identify which result is truly reactive; however, this conservative default may unintentionally restrict organ utilization and conflict with evolving transplant practices that support the safe use of organs from viremic donors.

Despite widespread use of multiplex NAT platforms, the frequency, characteristics, and clinical consequences of invalid discriminatory NAT results in deceased organ donors have not been systematically evaluated. The objective of this study was to characterize the incidence of invalid discriminatory NAT results in deceased organ donors, evaluate associated serologic patterns and operational delays, and assess their impact on organ utilization in the United States.

## 2 Methods

NAT results from 97 780 deceased organ donors were analyzed retrospectively for this study. Donor specimens were collected and tested throughout the United States and tested within 11 different laboratories. Result data were collected from laboratories within a single network. Donor specimens were submitted to the laboratories from 38 OPOs.

Food and Drug Administration (FDA)‐approved donor screening tests were used to determine donor eligibility. NAT was performed using the Procleix Ultrio Elite assay (Grifols Diagnostic Solutions Inc., Emeryville, CA). The workflow diagram is depicted in Figure 1. The sensitivity of the Procleix Ultrio Elite assay and the Procleix Ultrio Elite HIV, HCV, and HBV discriminatory assays has been demonstrated for specimens with HIV‐1 viral RNA concentrations equal to or greater than 100 copies/mL, HIV‐2 viral RNA concentrations equal to or greater than 100 copies/mL, HCV viral RNA concentrations equal to or greater than 30 IU/mL, or HBV viral DNA concentrations equal or greater than 6 IU/mL. Samples with less than these concentrations may not yield reproducible results [7].

**FIGURE 1:**
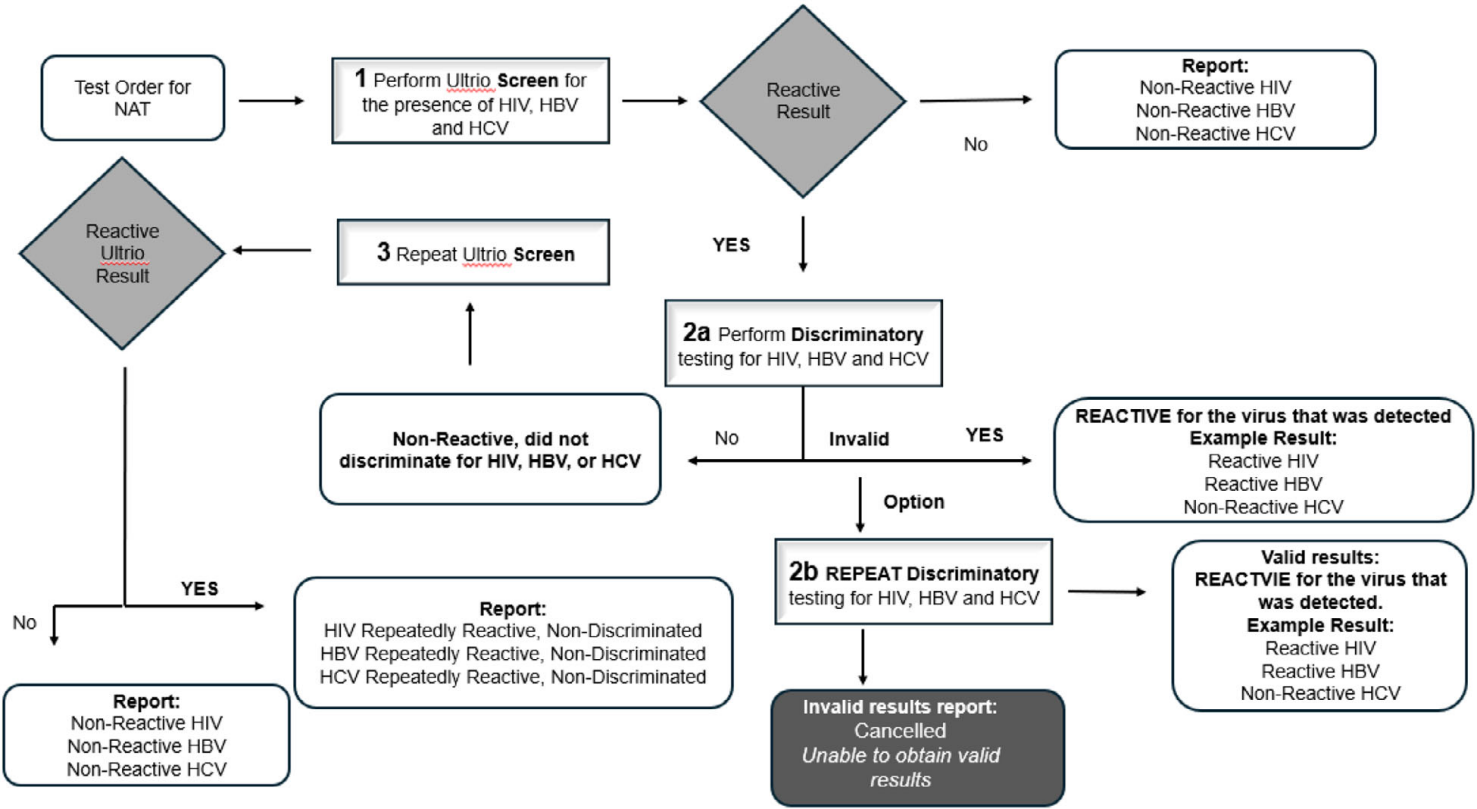
NAT testing algorithm. Nucleic acid test algorithm that was used for deceased donor testing. When discriminatory results are not obtained, the laboratory repeats testing in an effort to obtain a result.

In the Procleix Ultrio Elite HIV, HCV, and HBV discriminatory assays, specimens with an internal control signal greater than 475 000 relative light units (RLU) are invalidated by the software, and the reactive status of the donor cannot be assessed [3].

Hepatitis B core total (anti‐HBc) and HCV antibodies (anti‐HCV) were detected using either manual Ortho ELISA assays (ORTHO HCV Version 3.0, Ortho Clinical Diagnostics, Raritan, NJ, USA) or the Abbott Alinity s platform (Alinity s Anti‐HCV and Anti‐HBc, Abbott Laboratories, Abbott Park, IL, USA). HIV‐1/2 Plus O antibodies and hepatitis B surface antigen (HBsAg) were measured using either manual Bio‐Rad ELISA assays (GS HIV‐1/HIV‐2 PLUS O and GS HBsAg EIA 3.0, Bio‐Rad Laboratories, Redmond, WA, USA) or the Abbott Alinity s platform (HIV Ag/Ab Combo and HBsAg Reagent Kit, Abbott Laboratories, Abbott Park, IL, USA).

Results were queried using data from the laboratories’ Laboratory Information Management System between January 2020 and May 2025. Statistical analysis was performed to compare RLU values using the Mann–Whitney *U* test. No AI tool were used for any part of this manuscript.

## 3 Results

### 3.1 Incidence of Invalid dNAT Results

NAT is essential for evaluating the safety of deceased organ donors; however, in rare instances, NAT results could not be fully resolved. Among 97 780 deceased donors screened during this study period, invalid discriminatory results occurred in 0.027% of cases. A total of 27 donors had failed discriminatory NAT results (Table 1; see also Figure 1 for the testing algorithm for this assay). Invalid dNAT results occurred more frequently for donors with likely HCV infection (*n* = 22) than for HBV infection (*n* = 6).

**TABLE 1:** Incidence of invalid discriminatory NAT results.

dNAT and serology results						
dHCV+ and invalid dHIV and/or dHBV NAT			dHBV+ and invalid dHIV and/or dHCV NAT			
Case #	dHCV	Anti‐HCV	Case #	dHBV	sAg	Core total Ab
1	+	+	22	+	+	+
2	+	+	23	+	+	+
3	+	−	24	+	+	+
4	+	+	25	+	NT	NT
5	+	+	26	+	+	+
6	+	+	27	+	NT	NT
7	+	+				
8	+	+				
9	+	+				
10	+	+				
11	+	+				
12	+	NT				
13	+	NT				
14	+	+				
15	+	−				
16	+	+				
17	+	−				
18	+	+				
19	+	+				
20	+	+				
21	+	+				
22	+	+				

Abbreviation: NT = testing not performed.

Cases presented in which discriminatory NAT results could not be obtained with the relevant associated serological test results.

The RLU values from the discriminatory assays were analyzed. RLU values obtained from cases with reactive results for either HBV or HCV (and nonreactive for HIV and either HCV or HBV) were compared to values from cases in which there were reactive HBV or HCV results (and invalid HIV and either HCV or HBV). No statistical differences in RLU values were observed. This indicates that RLU values cannot be used to gain insight into potential viral load.

### 3.2 Serologic Patterns Among Affected Donors

Among donors with HCV‐*reactive* discriminatory NAT and *invalid* HIV and/or invalid HBV NAT results (*n* = 22), 17 donors with available serologic testing were anti‐HCV‐positive (Table 1).

In five cases, discriminatory NAT results were *reactive* for HBV and invalid for HIV and/or HCV NAT (Table 1; Cases 22–27). In four of six cases, serologic results demonstrated findings consistent with HBV infection (surface antigen positive/core total antibody positive).

There was one case (Case 22) in which reactive discriminatory NAT results were obtained for both HBV and HCV, but invalid for HIV NAT results. Serology results were consistent with infection with HCV and HBV. Per the manufacturer's package insert instructions, invalid results for all three viruses were reported.

### 3.3 Repeat Testing and Turnaround Time

In many cases, testing was repeated to attempt to obtain valid discriminatory NAT results for all three viruses, including testing using alternative specimen types (plasma or serum). Despite repeated attempts, discriminatory NAT results could not be resolved in any case. These repeated testing efforts delayed the issuance of infectious disease results required by the Organ Procurement and Transplantation Network (OPTN) for organ allocation in the United States. While standard donor infectious disease results are typically reported within eight hours of sample receipt, additional case review processes, including conversations with OPOs regarding sample quality, donor clinical stability, and the availability of a new specimen from a different draw time, as well as repeat testing, introduced further delays. Each case required individualized assessment given the unique donor circumstances. As a result, these combined efforts added a median 22 h (IQR: 17.0–28.6) to reporting times, further impacting the timeliness of donor evaluation (Figure 2).

**FIGURE 2:**
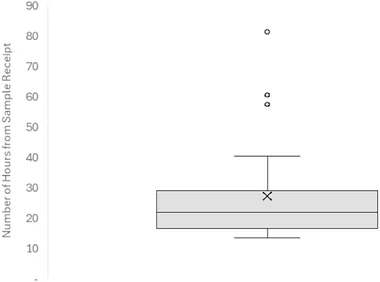
Turnaround times for the final test report after attempts to obtain valid discriminatory NAT results. Extended turnaround times from sample receipt to final report were analyzed. Report times were delayed while attempting to obtain valid results, which did not occur. Median time to final report issuance was 22 h.

### 3.4 Organ Utilization Outcomes

In some instances, clinicians proceeded with transplantation despite unresolved discriminatory NAT results. Organ allocation was provided for 21 cases. Of these cases, organs were only successfully placed in five cases (Table 2). However, in 30% of cases (*n* = 6), organs were not utilized, excluding one case in which the donor tested positive for COVID‐19.

**TABLE 2:** Organs placed despite invalid NAT results.

Case number	Kidneys	Heart	Lung	Liver
1	X	X	X	X
2	X	X^a^	X	X^b^
4	X^c^	X^c^	X^c^	X^c^
6	**✓**	**✓**	X	**✓**
14	**✓**	**✓**	X	**✓**
15	**✓**	**✓**	X	**✓**
16	X	X	X	X
17	**✓**	**✓**	**✓**	**✓**
18	X	X	X	X
19	X	X	X	X
20	**✓**	X	X	X
21	X	X	X	X

^a^ Donor had a pacemaker.

^b^ Ruled out in the operating room.

^c^ Donor tested positive for Covid‐19 and organs were not used.

In two donor cases of HCV‐reactive discriminatory NAT with invalid HIV and HBV results, there was sufficient time to perform quantitative PCR (qPCR) testing prior to allocation (Cases 14 and 15), which confirmed the presence of HCV viremia.

Transplant centers evaluate multiple factors when determining organ suitability, and their risk tolerance and decision‐making thresholds vary widely across the country. While it is difficult to quantify the exact impact, the absence of NAT results clearly introduces uncertainty and can significantly influence transplant centers’ willingness to accept an organ.

## 4 Discussion

This study identifies rare but clinically consequential cases in which discriminatory NAT results cannot be resolved in deceased organ donors. Although these events occurred infrequently, their impact on organ utilization can be substantial. At present, only qualitative NAT results are provided for organ allocation purposes, limiting the ability of transplant teams to contextualize unresolved or invalid results. In time‐sensitive allocation scenarios, this scenario complicates decision‐making and may contribute to unnecessary organ discard.

These findings bring to light the uncertainty of the deceased organ donor community's understanding of what to do with such results and the clinical implications of these results. When discriminatory NAT results cannot be obtained, several negative consequences arise. First, OPOs may classify donors as reactive for HIV, HBV, and HCV by default, which can lead to unnecessary exclusion of otherwise suitable organs and directly reduce organ availability. Second, repeated testing prolongs turnaround times for infectious disease reporting, which can render organs unsuitable due to time constraints. Third, unresolved NAT results introduce clinical uncertainty, prompting conservative decision‐making even in an era when effective antiviral therapies support safe transplantation from viremic donors. Finally, repeated testing imposes additional operational burdens on laboratories without necessarily resolving the underlying issue. Collectively, these factors limit transplant opportunities, delay organ allocation, and reduce the overall efficiency of the deceased donor transplantation process.

Quantitative viral testing may provide critical additional context in cases of invalid discriminatory NAT results, particularly when high viral loads are suspected of interfering with assay performance. Access to rapid, STAT qPCR testing in the deceased donor setting could enable more precise characterization of viremia, allowing transplant teams to differentiate true infection from assay‐related limitations. In time‐sensitive allocation scenarios, the availability of expedited quantitative results may reduce clinical uncertainty, support informed risk assessment, and ultimately improve organ utilization. Providing guidance for the inclusion of quantitative assay results to override invalid discriminatory NAT results could support more informed risk assessment and improve the efficiency of organ utilization in the United States. Although access to STAT qPCR is limited in many donor laboratories, selective availability or centralized reference testing may improve risk stratification in unresolved cases.

Interestingly, no cases were identified in which discriminatory NAT testing was reactive for HIV without corresponding HBV or HCV results. This observation suggests that high viral loads of HBV or HCV may interfere with TMA‐based discriminatory assays, although this mechanism cannot be confirmed in the present study. High titers of non‐target analytes may produce invalid results in individual Procleix Ultrio Elite discriminatory assays, and the assay manufacturer recommends alternate testing methods [7]. However, such diagnostic assays are often unavailable in STAT deceased donor laboratory settings.

Components of blood products, such as heparin, hemoglobin, or immunoglobulins, can inhibit the enzymes used in detection assays, leading to invalid or indeterminate results. Correlation between discriminatory NAT results and donors who received transfusion products was reviewed. There were no trends with the 27 cases presented.

This study has several limitations. Quantitative viral load testing was not available, preventing direct confirmation of viral interference. In the instances in which this testing was performed, samples were flown to a reference laboratory. In addition, the low incidence of invalid discriminatory NAT results limited formal statistical comparisons.

Improved transparency in the reporting of invalid NAT results within UNOS DonorNet is essential to support informed clinical decision‐making. Currently, the lack of standardized visibility into these results may obscure the true virologic profile of the donor, leading to overly conservative risk assessments. Providing clear annotation of invalid discriminatory NAT results, along with associated serologic (and available supplemental testing data), would allow transplant clinicians to better contextualize infection risk. Implementation of allowing the reporting of invalid NAT results would necessitate clear operational algorithms, standardized guidance, and targeted educational efforts to support both OPO and transplant center personnel. Enhanced reporting could facilitate more nuanced interpretation of donor status and help mitigate unnecessary organ discard driven by uncertainty. In practice, allocation‐related changes are typically introduced through structured processes led by OPTN, which include coordinated education, policy guidance, and ongoing monitoring of key performance metrics such as organ utilization and discard rates.

In conclusion, while invalid discriminatory NAT results are uncommon, they may represent a barrier to honoring donor intent and maximizing organ recovery. These findings underscore the need for the reporting of invalid results within the US allocation system and improved access to alternate confirmatory testing to support informed clinical decision‐making and reduce unnecessary organ discard.

## Author Contributions

**Sara Dionne**: conceptualization, writing – original draft, writing – review and editing, visualization, supervision. **Brenden O'neale**: formal analysis. **Chris Curran**: data curation. **Sean Vanslyck**: conceptualization, writing – review and editing, data curation. **Kaitlin Logue**: data curation, formal analysis, writing – review and editing, visualization. **Hillary Akana**: methodology, writing – review and editing, formal analysis, project administration, data curation, visualization, investigation.

## Funding

The authors have nothing to report.

## Ethics Statement

Ethical approval was not sought for the present study because the data presented here were collected retrospectively; donors consented at the time of donation to be screened for infectious diseases as part of the routine donation process. This data is not sensitive and has no risk for compromising the identity/safety of patients.

## Conflicts of Interest

The authors declare no conflicts of interest.

## Supporting information




**Supporting information**: Visual Abstract
